# Evaluation of pleiotropic effects among common genetic loci identified for cardio-metabolic traits in a Korean population

**DOI:** 10.1186/s12933-016-0337-1

**Published:** 2016-02-01

**Authors:** Yun Kyoung Kim, Mi Yeong Hwang, Young Jin Kim, Sanghoon Moon, Sohee Han, Bong-Jo Kim

**Affiliations:** Division of Structural and Functional Genomics, Center for Genome Sciences, National Institute of Health, Centers for Disease Control and Prevention, Cheongju-si, Chungcheongbuk-do 28159 South Korea

**Keywords:** Genome-wide association study, Lipids, Cardio-metabolic traits, Pleiotropy, 12q24.12

## Abstract

**Background:**

The genetic contribution to complex diseases or traits, including cardio-metabolic traits, has been elucidated recently by large-scale genome-wide association studies. These genome-wide association studies have indicated that most pleiotropic loci contain genes associated with lipids. Clinically, lipid related abnormalities are strongly associated with other diseases such as type 2 diabetes, coronary artery disease and hypertension. The aim of this study was to evaluate the shared genetic background of lipids and other cardio-metabolic traits.

**Methods:**

We conducted meta-analyses of the association between 157 published lipid-associated loci and 10 cardio-metabolic traits in 14,028 Korean individuals genotyped using the Exome chip (Illumina HumanExome BeadChip). We also examined whether the pleiotropic effects of such loci constituted independent (i.e., biological) pleiotropy or mediated pleiotropy in these metabolic pathways.

**Results:**

Eighteen lipid-associated loci were significantly associated with one of six cardio-metabolic traits after correction for multiple testing (*P* < 3.70 × 10^−4^). Region 12q24.12 had pleiotropic effects on fasting plasma glucose, blood pressure and obesity-related traits (body mass index and waist-hip ratio) independent of its effects on the lipid profile. Lipid risk scores, calculated according to whether or not subjects carried the risk allele for lipid traits, were significantly associated with fasting plasma glucose, blood pressure and obesity-related traits.

**Conclusions:**

The 12q24.12 region showed ethnic-specific genetic pleiotropy among cardio-metabolic traits in this study. Our findings may help to account for molecular mechanisms based on shared genetic background underlying not only dyslipidemia, but also cardiovascular disease and type 2 diabetes.

**Electronic supplementary material:**

The online version of this article (doi:10.1186/s12933-016-0337-1) contains supplementary material, which is available to authorized users.

## Background

The etiology of cardiovascular disease (CVD) is complex, as both genetic and environmental factors contribute [1]. Common risk factors for CVD are cardio-metabolic traits such as lipids, blood pressure, obesity and diabetes [2]. Of these, lipid-related abnormalities are strongly associated with type 2 diabetes (T2D)-related traits and hypertension [3]. For example, treatment of dyslipidemia can reduce the incidence of T2D [4], and circulating levels of triglycerides (TG) and high-density lipoprotein (HDL) cholesterol are predictors of T2D [3]. However, although there are strong correlations between lipid-related abnormalities and cardio-metabolic traits at the clinical level, the genetic and molecular mechanisms underlying the link between lipids and other cardio-metabolic traits remain unclear.

Genetic contributions to complex diseases or traits, including cardio-metabolic traits, have been analyzed by large-scale genome-wide association studies (GWASs) [5–7]. To date, more than 157 loci have been associated with lipids [5]. Some of these loci are pleiotropic, that is, they also showed significant association with other cardio-metabolic traits [8]. The forms of pleiotropy are important to distinguish because they have different implications for disease risk and pathogenesis. Independent pleiotropy is considered as genuine pleiotropy, and refers to a genetic variant that affects more than one trait independently [9]. In contrast, mediated pleiotropy exists when one genetic variant has an effect on a trait that in turn affects another trait [10].

In this study, we aimed to examine the pleiotropic effects of published lipid-associated loci on 10 cardio-metabolic traits in a Korean population using data from 14,028 individuals directly genotyped by the Exome chip (Illumina HumanExome BeadChip). We also explored whether or not the observed pleiotropic associations were independent or mediated, and tested the hypothesis that the effects of associations between lipid loci and other cardio-metabolic traits were controlled by lipids. By making lipid risk scores, we examined the cumulative genetic effect of lipid-associated loci on other cardio-metabolic traits. Our results suggest the presence of shared genetic backgrounds and biological pathways for lipids and other risk factors for CVD.

## Methods

### Study participants

We performed Exome chip genotyping of 14,616 study participants from three population-based cohorts that are comprised in the Korean Genome Epidemiology Study (KoGES). We selected 7981 participants from the Ansung and Ansan population-based cohort (the Korea Association REsource (KARE) project), 3448 participants from the Health Examinee (HEXA) cohort and 3187 participants from Cardio Vascular Disease Association Study (CAVAS). All participants were aged between 40 and 69 years. More detailed descriptions of these three cohorts are available in published articles [11–13]. All participants provided written informed consent, and this study was approved by the ethical committee of our institute (Korea Centers for Disease Control and Prevention Institutional Review Board).

### Phenotype determination

Blood levels of glycemic traits [fasting plasma glucose (FPG), glycated hemoglobin (HbA1c)] and lipids [TG, total cholesterol (TC), and HDL] were measured in plasma (FPG), whole blood (HbA1c) and serum (lipids), respectively, using standard enzymatic methods from fasting-individuals. If the TG level was <400 mg/dl, the concentration of low-density lipoprotein (LDL) cholesterol was calculated using Friedewald’s formula [14]. Blood pressure (BP) was measured using a standard mercury sphygmomanometer after participants had been in a sitting position for at least 5 min. The mean of two measures (left and right arm) was taken as the BP. If participants were taking antihypertensive agents, BP values were adjusted for analyses by adding 10 mmHg to systolic BP (SBP) and 5 mmHg to diastolic BP (DBP) [7]. Body mass index (BMI) and waist-hip ratio (WHR) were defined as weight (in kilograms) divided by the square of height (in meters) and the ratio of waist to hip circumferences (in centimeters), respectively.

Individuals taking lipid lowering or anti-diabetes medication and individuals with FPG ≥ 126 mg/dl and HbA1c ≥ 6.5 % were excluded from the analyses to obtain only non-diabetic individuals.

### Exome chip

The Illumina HumanExome BeadChip is a genotyping array that includes variants represented by ~250,000 single nucleotide polymorphisms (SNPs) selected from exome and whole-genome sequences in ~12,000 individuals [15]. This chip is focused on protein-altering variants (non-synonymous, stop and splice variants) and represents diverse populations and multiple complex traits [16, 17]. More detailed explanations about the contents of the Exome chip can be found at the Exome chip design web page (http://genome.sph.umich.edu/wiki/Exome_Chip_Design).

### Genotyping and quality control

All 14,616 study participants were genotyped using the Illumina HumanExome BeadChip v1.1 (variants = 242,901 SNPs). For genotype calling, each genotype was automatically clustered by the Illumina GenomeStudio v2011.1 software [18]. To improve the accuracy of variant calling, manual re-clustering and visual inspection were performed for genotypes based on the CHARGE clustering method [15]. After genotype calling, variants with completely missing (n = 1431), monomorphic SNPs (n = 162,803), Hardy–Weinberg equilibrium values of *P* < 1.0 × 10^−6^ (n = 1184) or genotype call rates of < 0.95 (n = 11) were excluded for quality control. Samples with a genotype call rate of < 99 % (n = 43), sex discrepancy (n = 51) or cryptic relatedness (n = 487), and withdrawal of participation (n = 7) were also removed for sample quality control.

After genotype and sample quality control, 77,472 SNPs and 14,028 samples (7524 from KARE, 3436 from HEXA and 3068 from CAVAS) were available for further analyses. The number of SNPs with different minor allele frequencies is summarized in Additional file 1: Table S1.

### Selection of lipid-associated SNPs for analyses

We focused on 157 loci that have been identified previously in large GWAS on lipids with genome-wide significance (*P* < 5 × 10^−8^) [5]. Different lipids can have different best-associated SNPs in the same locus. After selection of the most strongly associated SNPs for each locus, 157 regions were mapped to 129 genes and 135 SNPs [minor allele frequency (MAF) > 0.001, equivalent to a minor allele count ≥ 5 in each cohort].

### Calculation of lipid risk score

To test the combined effects of lipid SNPs on other cardio-metabolic traits, we calculated two types of risk score, an unweighted risk score and a weighted risk score, for each SNP and each individual based on 49 lipid-related SNPs that were replicated in this study. The list of SNPs is provided in Additional file 1: Table S3. The unweighted risk score was determined as the total number of risk alleles per individual, and the weighted risk score was calculated by weighting the risk alleles by their estimated effect size based on our data in this study [19]. Associations between lipid risk scores and cardio-metabolic traits were evaluated based on a linear regression model using the R program (version 2.15.3; http://www.r-project.org/). In this program, a linear model (function “lm” in the “stats” package) is constructed that includes variables such as lipid risk scores and cardio-metabolic traits. For example, when we choose a model with three variables, the script is as follows; > fit <− lm (y ~ x1 + x2 + x3, data = riskscore), > summary (fit). Age, sex and lipids (TG, TC, LDL and HDL) were included in the analyses as covariates.

### Statistical analyses

Single-variant association analysis was conducted between 135 SNPs and 10 cardio-metabolic traits (TG, TC, LDL, HDL, SBP, DBP, FPG, HbA1c, BMI and WHR) using a linear regression model via EPACTS v3.2.4 (http://genome.sph.umich.edu/wiki/EPACTS) and PLINK 1.07 (http://pngu.mgh.harvard.edu/~purcell/plink/) programs [20]. The main command of association analysis is “--test q.linear” and “--linear” to each program, respectively. To address the issue of multiple testing, a robust threshold of association significance (⍺ = 0.05/135 for each trait, *P* < 3.7 × 10^−4^) was used based on the Bonferroni correction. Phenotypes used in the analyses were approximately normally distributed, and age and sex were incorporated into the analyses as covariates. Meta-analyses of association results from each cohort were performed with the inverse variance method using the METAL program (http://genome.sph.umich.edu/wiki/METAL) [21].

To evaluate and clarify the type of pleiotropy (independent or mediated) for three pleiotropic SNPs located on region 12q24.12, additional analyses were performed including adjustment of lipids (TC, TG, LDL and HDL) for other cardio-metabolic traits to examine whether or not any association occurred independently of lipids effects.

### Protein–protein interactions

To search for functional interactions among genes, protein–protein interactions were performed using Wiki-Pi (http://severus.dbmi.pitt.edu/wiki-pi/) [22]. This tool provides information about one or both proteins in the interaction based on automatically updated annotations of individual proteins from databases such as Gene Ontology, Kyoto Encyclopedia of Genes and Genomes, REACTOME, Pubmed2ENSEMBL and DrugBank.

## Results

One hundred and fifty-seven lipid-associated loci, mapping to 129 genes and 135 SNPs (MAF > 0.001, equivalent to a minor allele count ≥ 5) in our directly genotyped Exome chip data, were selected for analysis. All loci had a genome-wide significance *P* value (*P* < 5 × 10^−8^) according to the Global Lipids Genetics Consortium [5]. The associations between each of the 135 SNPs and each of the 10 cardio-metabolic traits (TG, TC, HDL, LDL, SBP, DBP, FPG, HbA1c, BMI and WHR) were examined in 14,028 Korean individuals from three cohorts based on Exome chip genotyping. Baseline characteristics of the study participants are summarized in Additional file 1: Table S2.

### Genetic effects of previously implicated lipid-associated SNPs

**Fig. 1:**
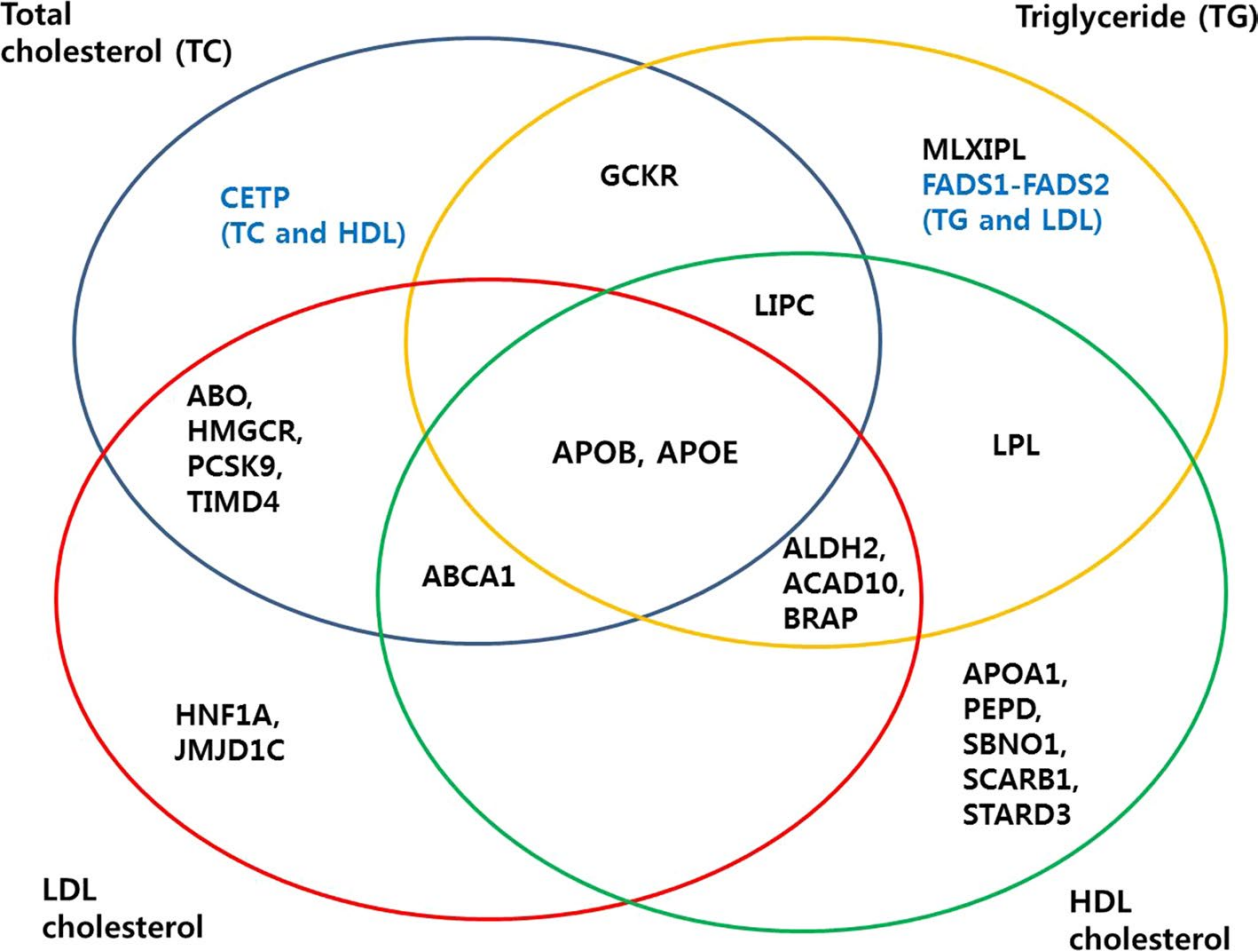
Overlap of loci associated with different lipids. This *Venn diagram* illustrates the name of lipid-associated loci that replicated in our study (*P* < 3.70 × 10^−4^)

In the meta-analyses, 49 loci with new lead SNPs were significantly associated with one of four lipid traits (10 loci associated with TC, 11 with TG, 14 with HDL and 14 with LDL) after correction for multiple testing (*P* < 3.70 × 10^−4^) (Additional file 1: Table S3). Most signals were associated with two or more lipid traits. The overlap of loci associated with different lipid traits is shown in Fig. 1. Among the 49 replicated SNPs, 22 (45 %) were located in exonic regions and, of these, 18 (82 %) were non-synonymous variants. Several non-synonymous variants, which were located on *PCSK9*, *CETP* and *APOA1* associated with TC or HDL and were novel protein-coding variants that had not been revealed in the same loci in previous studies.

### Association between lipid-associated SNPs and other cardio-metabolic traits

**Fig. 2:**
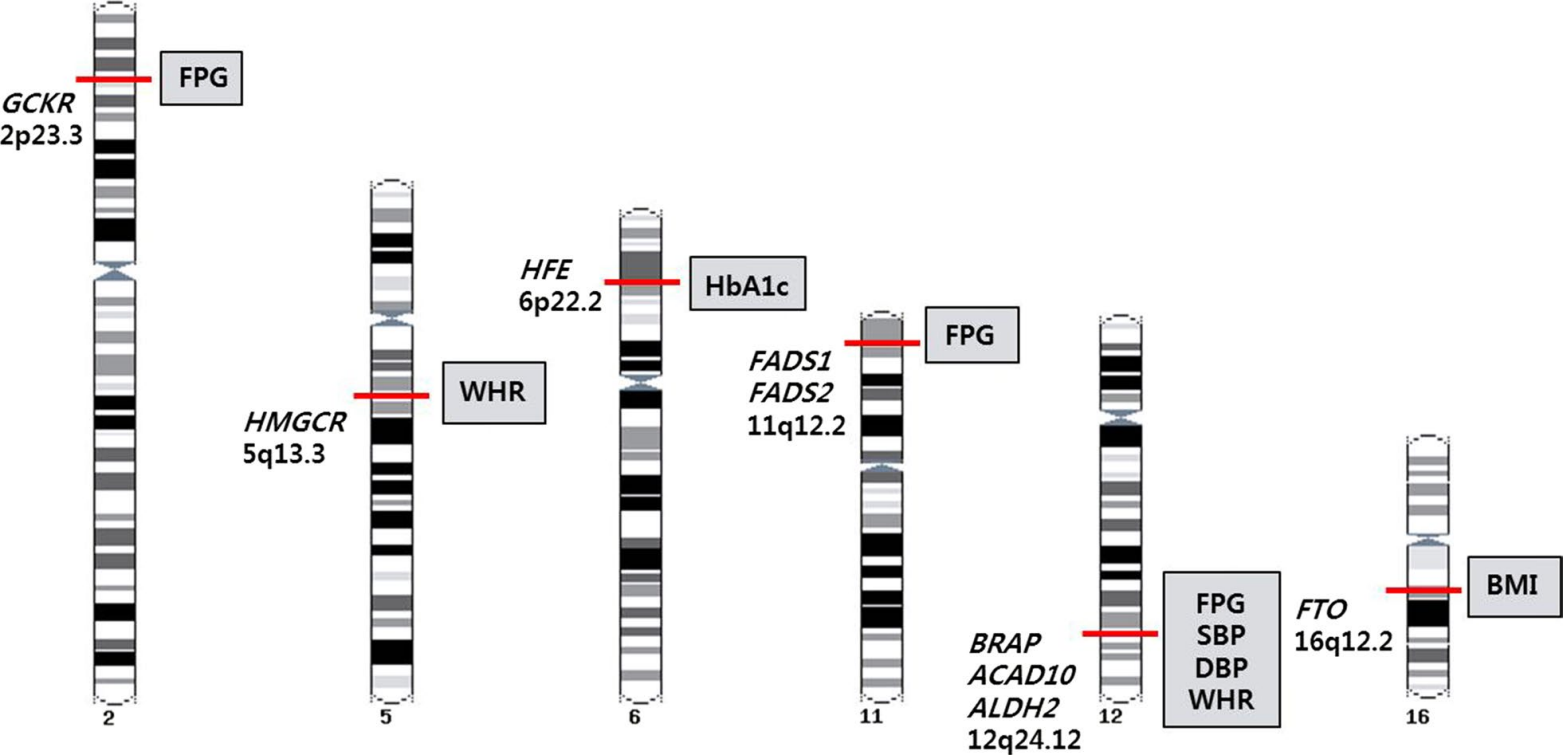
Summary of meta-analyses for associations between previously implicated lipid loci and cardio-metabolic traits. There are genes and genetic loci on chromosomes associated with cardio-metabolic traits that reached the statistical significance (*P* < 3.70 × 10^−4^) after correction for multiple testing in this study

Eighteen out of 135 SNPs were associated with other cardio-metabolic traits such as FPG, HbA1c, BMI, WHR, SBP and DBP after correction for multiple testing (*P* < 3.70 × 10^−4^) (Table 1; Fig. 2). Our findings replicated those of previous GWASs for five genes (*GCKR*, *FADS1* and *FADS2* for FPG, *HFE* for HbA1c and *FTO* for BMI) and also identified novel associations for 13 genes (three for FPG, four for WHR, three for SBP and three for DBP). Three associations were observed with one or more of four cardio-metabolic traits (FPG, WHR, SBP and DBP) within the genes *BRAP*, *ACAD10* and *ALDH2* in the 12q24.12 region (Table 1; Fig. 2). These associations had not been reported in previous GWASs. The three SNPs were composed of two non-synonymous and one intronic variant and had same direction of effect for each trait. All three SNPs showed monomorphic ethnic differences in allele frequency from European and South Asian descent in comparison with allele frequency from our data (Korean individuals, average MAF = 0.161) and East Asian descent (average MAF = 0.175; calculated using data from the 1000 Genomes Project, http://www.1000genomes.org/) (Additional file 1: Table S4).

**Table 1:** Results of meta-analyses for associations between lipid loci and cardio-metabolic traits

Category	Trait	CHR	SNP	Position	Gene	Function	A1/A2	MAF	Effect size (beta ± s.e.)	*P*_combined_	*P*_het_ (Q)
Glycemic traits (n = 13,378)	FPG	2	rs780093	27742603	*GCKR*	Intronic	G/A	0.456	0.73 ± 0.13	6.26 × 10^−9^	0.72 (0.64)
		11	rs174547	61570783	*FADS1*	Intronic	G/A	0.319	−0.70 ± 0.13	1.71 × 10^−7^	0.94 (0.12)
		11	rs174570	61597212	*FADS2*	Intronic	A/G	0.319	−0.71 ± 0.13	1.09 × 10^−7^	0.97 (0.07)
		12	rs3782886	112110489	*BRAP*	Nonsynonymous	G/A	0.167	−0.90 ± 0.17	7.21 × 10^−8^	0.67 (0.79)
		12	rs11066015	112168009	*ACAD10*	Intronic	A/G	0.158	−0.92 ± 0.17	5.93 × 10^−8^	0.74 (0.60)
		12	rs671	112241766	*ALDH2*	Nonsynonymous	A/G	0.157	−0.96 ± 0.17	2.00 × 10^−8^	0.80 (0.44)
	HbA1c	6	rs1799945	26091179	*HFE*	Nonsynonymous	G/C	0.045	−0.06 ± 0.01	4.57 × 10^−5^	–
Blood pressure (n = 10,310)	SBP	12	rs3782886	112110489	*BRAP*	Nonsynonymous	G/A	0.167	−1.34 ± 0.31	1.50 × 10^−5^	0.04 (4.15)
		12	rs11066015	112168009	*ACAD10*	Intronic	A/G	0.158	−1.53 ± 0.32	1.33 × 10^−6^	0.03 (4.50)
		12	rs671	112241766	*ALDH2*	Nonsynonymous	A/G	0.157	−1.49 ± 0.32	2.72 × 10^−6^	0.05 (4.01)
	DBP	12	rs3782886	112110489	*BRAP*	Nonsynonymous	G/A	0.167	−0.78 ± 0.20	1.02 × 10^−4^	0.49 (0.48)
		12	rs11066015	112168009	*ACAD10*	Intronic	A/G	0.158	−0.82 ± 0.21	7.12 × 10^−5^	0.41 (0.67)
		12	rs671	112241766	*ALDH2*	Nonsynonymous	A/G	0.157	−0.80 ± 0.21	1.09 × 10^−4^	0.47 (0.52)
Obesity (n = 13,378)	BMI	16	rs1421085	53800954	*FTO*	Intronic	G/A	0.125	0.29 ± 0.06	1.84 × 10^−7^	0.70 (0.71)
	WHR	5	rs3846662	74651084	*HMGCR*	Intronic	A/G	0.476	0.003 ± 0.001	3.69 × 10^−4^	0.29 (2.45)
		12	rs3782886	112110489	*BRAP*	Nonsynonymous	G/A	0.167	−0.004 ± 0.001	3.45 × 10^−5^	0.12 (4.32)
		12	rs11066015	112168009	*ACAD10*	Intronic	A/G	0.158	−0.005 ± 0.001	3.35 × 10^−6^	0.13 (4.14)
		12	rs671	112241766	*ALDH2*	Nonsynonymous	A/G	0.157	−0.005 ± 0.001	2.87 × 10^−6^	0.13 (4.10)

A threshold of association significance after correction for multiple testing is *P* < 3.70 × 10^−4^

*CHR* chromosome, *A1/A2* minor allele/major allele, *MAF* minor allele frequency, *n* number of participants, *SBP* systolic blood pressure, *DBP* diastolic blood pressure, *FPG* fasting plasma glucose, *HbA1c* Glycated hemoglobin, *BMI* body mass index, *WHR* waist-hip ratio; All of analyses were adjusted by age and sex. *s.e.* standard error, *P*_*combined*_ *P* value from meta-analyses using the results from three cohorts, *P*_*het*_ *P* value from test of heterogeneity, *Q* Cochrane’s Q value based on Chi squared statistics

### The pleiotropic effect of SNPs in region 12q24.12

**Fig. 3:**
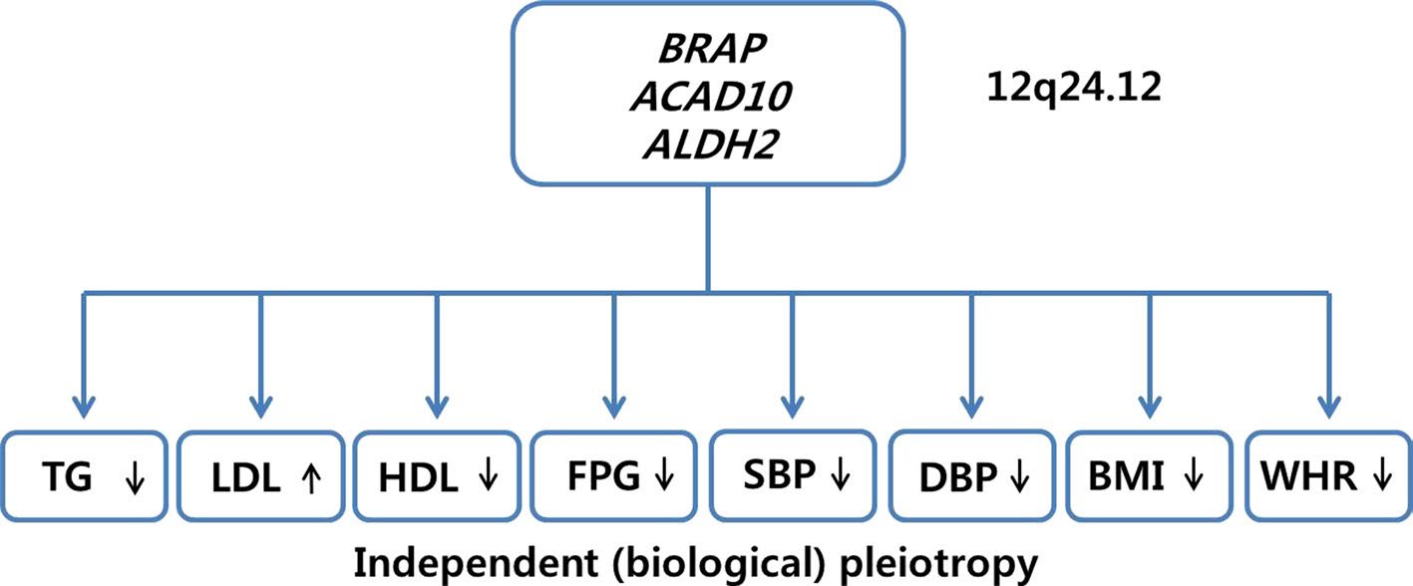
Pleiotropic effects on three genes (*BRAP*, *ACAD10* and *ALDH2*) in the 12q24.12 region. This region was associated independently with each cardio-metabolic trait according to additional analyses including adjustments. The *black arrows* represented the direction of genetic effects on each trait

The pleiotropic effect of region 12q24.12 was identified for three SNPs (rs3782886 on *BRAP*, rs11066015 on *ACAD10* and rs671 on *ALDH2*) on lipids and other cardio-metabolic traits (Table 2). These SNPs were associated with three lipid traits (TG, HDL and LDL), SBP, DBP, FPG, BMI and WHR (*P* < 0.05) and had the same direction of allelic effect for all cardio-metabolic traits (the linkage disequilibrium of these SNPs in our data, r^2^ = 0.988 between rs671 and rs11066015, r^2^ = 0.926 between rs671 and rs3782886) (Additional file 1: Figure S1). To identify the type of pleiotropy, adjusted association analyses were repeatedly performed across the eight cardio-metabolic traits. Mediated analyses showed that the association between rs671 and the three lipid traits (TG, HDL and LDL) remained significant after adjustments for all other cardio-metabolic traits (Table 3; Fig. 3; Additional file 1: Table S5). In addition, the association between rs671 and FPG, SBP, DBP, BMI and WHR remained significant after adjustments for the three lipid traits, suggesting that rs671 on *ALDH2* is independently associated with each cardio-metabolic trait (Table 3; Fig. 3; Additional file 1: Table S5). The adjusted associations of rs3782886 and rs11066015 with each cardio-metabolic trait showed the same effects as rs671 (data not shown).

**Table 2:** Pleiotropic effects of three SNPs located in the 12q24.12 region

Traits	n	rs671, *ALDH2*		rs11066015, *ACAD10*		rs3782886, *BRAP*	
		Effect size (beta ± s.e.)	*P*	Effect size (beta ± s.e.)	*P*	Effect size (beta ± s.e.)	*P*
Lipids							
TC	13,378	−0.03 ± 0.59	0.9547	−0.03 ± 0.59	0.9633	−0.26 ± 0.58	0.6502
TG	13,378	−7.60 ± 1.72	9.47 × 10^−6^	−7.28 ± 1.71	2.11 × 10^−5^	−6.93 ± 1.68	3.53 × 10^−5^
LDL	13,378	2.55 ± 0.53	1.53 × 10^−6^	2.54 ± 0.53	1.50 × 10^−6^	2.13 ± 0.52	3.80 × 10^−5^
HDL	13,378	−1.33 ± 0.18	1.20 × 10^−13^	−1.33 ± 0.18	7.61 × 10^−14^	−1.33 ± 0.17	2.97 × 10^−14^
Blood pressure							
SBP	10,310	−1.49 ± 0.32	2.72 × 10^−6^	−1.53 ± 0.32	1.33 × 10^−6^	−1.34 ± 0.31	1.50 × 10^−5^
DBP	10,310	−0.80 ± 0.21	1.09 × 10^−4^	−0.82 ± 0.21	7.12 × 10^−5^	−0.78 ± 0.20	1.02 × 10^−4^
Glycemic traits							
FPG	13,378	−0.96 ± 0.17	2.00 × 10^−8^	−0.92 ± 0.17	5.93 × 10^−8^	−0.90 ± 0.17	7.21 × 10^−8^
HbA1c	7524	0.003 ± 0.008	0.6858	0.006 ± 0.008	0.4870	0.004 ± 0.008	0.5924
Obesity							
BMI	13,378	−0.16 ± 0.05	2.14 × 10^−3^	−0.16 ± 0.05	2.10 × 10^−3^	−0.13 ± 0.05	1.06 × 10^−2^
WHR	13,378	−0.005 ± 0.001	2.87 × 10^−6^	−0.005 ± 0.001	3.35 × 10^−6^	−0.004 ± 0.001	3.45 × 10^−5^

*n* number of participants, *s.e.* standard error, *TG* Triglyceride, *TC* Total cholesterol, *LDL* Low density lipoprotein cholesterol, *HDL* high density lipoprotein cholesterol, *SBP* systolic blood pressure, *DBP* diastolic blood pressure, *FPG* fasting plasma glucose, *HbA1c* Glycated hemoglobin, *BMI* body mass index, *WHR* waist-hip ratio

All of analyses were adjusted by age and sex

**Table 3:** Evaluation of pleiotropic effect of 12q24.12 (*ALDH2*, rs671) genotypes

Trait	Effect size(beta ± s.e.)	*P* value after adjustment for age and sex	After adjustment for an additional covariate							
			TG		LDL		HDL		FPG	
			Effect size	*P* value	Effect size	*P* value	Effect size	*P* value	Effect size	*P* value
TG	−7.60 ± 1.72	9.47 × 10^−6^	–	–	−4.34 ± 1.18	2.44 × 10^−4^	−12.24 ± 1.64	8.41 × 10^−14^	−6.34 ± 1.68	1.58 × 10^−4^
LDL	2.55 ± 0.53	1.53 × 10^−6^	2.42 ± 0.54	7.47 × 10^−6^	–	–	2.76 ± 0.54	3.24 × 10^−7^	2.97 ± 0.56	9.78 × 10^−8^
HDL	−1.33 ± 0.18	1.20 × 10^−13^	−1.70 ± 0.18	1.80 × 10^−21^	−1.53 ± 0.19	8.14 × 10^−16^	–	–	−1.42 ± 0.19	8.88 × 10^−14^
FPG	−0.96 ± 0.17	2.00 × 10^−8^	−0.96 ± 0.19	2.00 × 10^−7^	−1.11 ± 0.18	1.85 × 10^−9^	−0.96 ± 0.19	2.38 × 10^−7^	–	–
SBP	−1.49 ± 0.32	2.72 × 10^−6^	−1.25 ± 0.33	1.37 × 10^−4^	−1.45 ± 0.33	1.26 × 10^−5^	−1.50 ± 0.33	5.68 × 10^−6^	−1.09 ± 0.34	1.14 × 10^−3^
DBP	−0.80 ± 0.21	1.09 × 10^−4^	−0.67 ± 0.21	1.41 × 10^−3^	−0.83 ± 0.21	1.03 × 10^−4^	−0.90 ± 0.21	2.35 × 10^−5^	−0.66 ± 0.22	2.35 × 10^−3^
BMI	−0.16 ± 0.05	2.14 × 10^−3^	−0.11 ± 0.05	2.90 × 10^−2^	−0.20 ± 0.05	1.45 × 10^−4^	−0.26 ± 0.05	3.86 × 10^−7^	−0.14 ± 0.05	1.14 × 10^−2^
WHR	−0.005 ± 0.001	2.87 × 10^−6^	−0.004 ± 0.001	2.81 × 10^−4^	−0.005 ± 0.001	1.92 × 10^−5^	−0.007 ± 0.001	1.48 × 10^−10^	−0.004 ± 0.001	3.54 × 10^−4^

Trait	Effect size (beta ± s.e.)	*P* value after adjustment for age and sex	After adjustment for an additional covariate							
			SBP		DBP		BMI		WHR	
			Effect size	*P* value	Effect size	*P* value	Effect size	*P* value	Effect size	*P* value
TG	−7.60 ± 1.72	9.47 × 10^−6^	−6.89 ± 1.94	3.80 × 10^−4^	−6.92 ± 1.93	3.45 × 10^−4^	−6.34 ± 1.71	2.14 × 10^−4^	−5.71 ± 1.71	8.68 × 10^−4^
LDL	2.55 ± 0.53	1.53 × 10^−6^	2.66 ± 0.61	1.21 × 10^−5^	2.73 ± 0.61	7.29 × 10^−6^	2.72 ± 0.54	3.79 × 10^−7^	2.57 ± 0.54	2.09 × 10^−6^
HDL	−1.33 ± 0.18	1.20 × 10^−13^	−1.39 ± 0.19	3.16 × 10^−13^	−1.40 ± 0.19	2.87 × 10^−13^	−1.54 ± 0.19	1.45 × 10^−16^	−1.47 ± 0.19	9.01 × 10^−15^
FPG	−0.96 ± 0.17	2.00 × 10^−8^	−0.84 ± 0.20	2.35 × 10^−5^	−0.87 ± 0.20	1.20 × 10^−5^	−0.91 ± 0.18	6.32 × 10^−7^	−0.93 ± 0.19	4.85 × 10^−7^
SBP	−1.49 ± 0.32	2.72 × 10^−6^	–	–	−0.38 ± 0.19	4.78 × 10^−2^	−1.16 ± 0.32	3.00 × 10^−4^	−1.07 ± 0.32	9.21 × 10^−4^
DBP	−0.80 ± 0.21	1.09 × 10^−4^	−0.07 ± 0.12	0.5869	–	–	−0.63 ± 0.20	2.08 × 10^−3^	−0.57 ± 0.21	5.26 × 10^−3^
BMI	−0.16 ± 0.05	2.14 × 10^−3^	−0.10 ± 0.06	8.72 × 10^−2^	−0.10 ± 0.06	8.47 × 10^−2^	–	–	−0.05 ± 0.05	0.2964
WHR	−0.005 ± 0.001	2.87 × 10^−6^	−0.004 ± 0.001	8.28 × 10^−4^	−0.004 ± 0.001	7.50 × 10^−4^	−0.003 ± 0.001	5.59 × 10^−4^	–	–

Total cholesterol was excluded from this analysis. n = 13,378 for all traits except SBP and DBP, where n = 10,310

*TG* Triglyceride, *LDL* low density lipoprotein cholesterol, *HDL* high density lipoprotein cholesterol, *FPG* fasting plasma glucose, *SBP* systolic blood pressure, *DBP* diastolic blood pressure, *BMI* body mass index, *WHR* waist-hip ratio, *s.e.* standard error

### Protein–protein interactions

To assess the physical interactions among the three pleiotropic genes in the 12q24.12 region, protein–protein interactions were tested using the Wiki-Pi database (http://severus.dbmi.pitt.edu/wiki-pi/) [22]. There were numerous direct protein–protein interactions for the three genes: 12 interactions for *ALDH2*, one interaction for *ACAD10* and 15 interactions for *BRAP*. According to this analysis, the three pleiotropic genes were functionally connected by one gene, *APP*, which encodes amyloid beta (A4) precursor protein (Additional file 1: Figure S2). This protein participated in several biological pathways and showed 1995 interactions with other proteins within Wiki-Pi data (Additional file 1: Table S6). In particular, *APP* has a role in the cholesterol metabolic process and in blood coagulation (Additional file 1: Table S6).

### Associations between lipid risk scores and other cardio-metabolic traits

To examine the cumulative genetic effect of the 49 replicated lipid SNPs on other cardio-metabolic traits such as FPG, SBP, DBP, BMI and WHR, a lipid risk score was calculated for TG, TC, HDL and LDL from different sets of SNPs, and two types of lipid risk score were calculated for each lipid. The weighted risk score for LDL was associated with all five cardio-metabolic traits (*P* < 0.05) (Table 4). The unweighted risk score of the four lipids was associated with FPG (*P* < 0.05) (Table 4).

**Table 4:** Pleiotropic association between lipid risk scores and cardio-metabolic traits

Lipid RS	SBP		DBP		FPG		BMI		WHR	
	Effect size	*P* value	Effect size	*P* value	Effect size	*P* value	Effect size	*P* value	Effect size	*P* value
LDL_RS	−0.24 ± 0.07	2.87 × 10^−4^	−0.12 ± 0.04	3.82 × 10^−3^	−0.17 ± 0.04	2.43 × 10^−6^	−0.02 ± 0.01	0.085	−0.0006 ± 0.0002	8.55 × 10^−3^
HDL_RS	−0.32 ± 0.07	9.05 × 10^−6^	−0.15 ± 0.05	1.54 × 10^−3^	−0.18 ± 0.04	7.17 × 10^−6^	−0.02 ± 0.01	0.165	−0.0004 ± 0.0002	0.113
TG_RS	−0.20 ± 0.07	5.57 × 10^−3^	−0.07 ± 0.05	0.103	−0.19 ± 0.04	1.55 × 10^−6^	−0.02 ± 0.01	0.160	−0.0007 ± 0.0002	2.07 × 10^−3^
TC_RS	0.08 ± 0.10	0.457	0.03 ± 0.07	0.623	0.26 ± 0.06	3.27 × 10^−6^	0.04 ± 0.02	6.21 × 10^−3^	0.0010 ± 0.0003	2.52 × 10^−3^
wLDL_RS	−0.04 ± 0.02	3.03 × 10^−2^	−0.04 ± 0.01	4.95 × 10^−3^	−0.06 ± 0.01	1.59 × 10^−7^	−0.02 ± 0.00	5.77 × 10^−9^	−0.0003 ± 0.0001	2.69 × 10^−5^
wHDL_RS	0.18 ± 0.05	4.42 × 10^−4^	0.17 ± 0.03	1.45 × 10^−7^	0.02 ± 0.03	0.396	0.07 ± 0.01	1.44 × 10^−18^	0.0017 ± 0.0002	8.11 × 10^−25^
wTG_RS	0.02 ± 0.01	0.070	0.01 ± 0.01	0.161	−0.01 ± 0.005	0.237	−0.003 ± 0.001	1.73 × 10^−2^	−0.00001 ± 0.00003	0.798
wTC_RS	−0.03 ± 0.02	0.183	−0.02 ± 0.01	0.125	−0.08 ± 0.01	2.32 × 10^−10^	−0.02 ± 0.004	1.55 × 10^−6^	−0.0002 ± 0.0001	8.87 × 10^−3^

*Lipid RS* lipid risk score, *LDL_RS* unweighted LDL risk score, *HDL_RS* unweighted HDL risk score, *TG_RS* unweighted TG risk score, *TC_RS* unweighted TC risk score, *wLDL_RS* weighted LDL risk score, *wHDL_RS* weighted HDL risk score, *wTG_RS* weighted TG risk score, *wTC_RS* weighted TC risk score, *SBP* systolic blood pressure, *DBP* diastolic blood pressure, *FPG* fasting plasma glucose, *BMI* body mass index, *WHR* waist-hip ratio

Effect size are shown as beta ± standard error. All of analyses were adjusted by age, sex and lipids

## Discussion

In this study, we performed meta-analyses of associations between 135 SNPs and 10 cardio-metabolic traits using Exome chip data from 14,028 Korean individuals to assess whether lipid-associated loci reported by the Global Lipids Genetics Consortium are also applicable to cardio-metabolic traits in this population. The 49 SNPs out of 135 SNPs that located lipid-associated loci were significantly replicated in these Korean participants after correction for multiple testing. Eighteen SNPs showed substantial pleiotropic effects on other cardio-metabolic traits that are risk factors for cardiovascular disease, such as lipids. We also reported a major pleiotropic effect of three genes in the 12q24.12 region on cardio-metabolic traits and confirmed the relevance of independent pleiotropic effect. Independent pleiotropy can occur at the regional level, whereby multiple variants in the same region are associated with different traits [10]. The physiological interrelation among these three genes was revealed by protein–protein interactions. They were connected by a mediator gene, *APP*, that participates in the lipid metabolic process [23].

At the 12q24.12 pleiotropic region, the functional non-synonymous variant rs671 on *ALDH2* which encodes the aldehyde dehydrogenase 2 family, has been reported to be associated with coronary artery diseases, blood pressure and alcohol consumption and is common in East-Asian populations in contrast to monomorphic in Europeans [7, 24]. One previous study provided evidence of positive selection at 12q24.12 by confirming the occurrence of a selective sweep in East-Asians by haplotype-based tests [7]. We predicted the functional effects, possibly damaging, potentially disease-causing biological effects, of the missense mutation at SNP rs671 using PolyPhen 2 and SIFT programs [25, 26]. In the current study, we established the genetic overlap of rs671 across eight cardio-metabolic traits, and first reported the association between this locus and FPG with genome-wide significance.

Two other SNPs in linkage disequilibrium (r^2^ > 0.9) with rs671 in Koreans, rs3782886 and rs11066015, showed the same genetic effects as rs671. Rs3782886 is a non-synonymous variant located on *BRAP*, which encodes BRCA1-associated protein, and rs11066015 is an intronic variant on *ACAD10*, which encodes acyl-CoA dehydrogenase family member 10. Previous studies have reported that *ACAD10* is associated with coronary artery disease and T2D, and it has been suggested that the effect of *ACAD10* on T2D might be mediated by disordered insulin resistance that is due to abnormal lipid oxidation [27, 28]. The two SNPs rs3782886 and rs11066015 were polymorphic only in individuals of East-Asian descent. This suggests that the pleiotropic associations described in our study may be specific to East-Asian individuals.

The three genes in the 12q24.12 region, *ALDH2*, *BRAP* and *ACAD10*, were functionally linked by a mediator gene, *APP*, that takes part in lipid-related metabolic processes (MIM104760). *APP* is a complex biological molecule that interacts with many types of receptors or proteins [29]. Mutations in *APP* have been implicated in Alzheimer’s disease and cerebroarterial amyloidosis [30]. Alzheimer’s disease is believed to be driven by the accumulation and deposition of the amyloid beta precursor protein that encoded by *APP* in brain region [23]. The abnormal accumulation or clearance of the amyloid beta precursor protein is regulated by cholesterol levels [23]. High LDL and low HDL is commonly found in patients with CVD and it has been shown to correlate with an increased risk of Alzheimer’s disease, with high risk being associated with advanced CVD [31]. As the three genes in 12q24.12 region is well known to association with CVD and lipid metabolism, the functional relation between *APP* and three genes in our study newly suggests that *APP* might play an important role in modulating CVD and dyslipidemia as well as Alzheimer’s disease.

An additional investigation was performed to reveal the combined genetic effect of lipid SNPs on other cardio-metabolic traits. This was conducted using lipid risk scores calculated by combination of the lipid SNPs implicated in this study. We were able to detect significant associations between unweighted or weighted lipid risk scores and cardio-metabolic traits. All four of the unweighted lipid risk scores were significantly associated with FPG. This provides compelling evidence that lipid risk SNPs have the potential to be used for identification of T2D risk. The finding that unweighted and weighted HDL risk scores were associated with SBP and DBP also suggests a shared genetic background between these traits. Our results showing an association between lipid risk scores and cardio-metabolic traits require replication in other ethnic groups.

### Future directions

In this study, we have presented lipid-related genetic variants with pleiotropic effects on other cardio-metabolic traits. Recent studies suggested that pleiotropic effects on complex traits may be widespread. Genetic variants associated with two or more traits, such as rs7138803 on *FAIM2* that related to obesity, T2D and myocardial infarction, rs8192673 on *PGC*-*1α* and rs1801282 on *PPAR*-*γ* associated with waist circumference and T2D, were revealed by previous studies [32–34]. The increased use of genetic information in clinical practice, has underscored the importance of understanding pleiotropy and its implications for genetic testing and personal genomics. A gene or multiple genes in a biological pathway that affect more than one trait present new chances and challenges for drug development. However, most studies have focused only on the detection of pleiotropic effects. Functionally characterizing identified variants through experiments and understanding the mechanisms of shared pathophysiology are necessary to establish causality. In addition, gene-environmental interactions (G × E) could provide insights the role of environmental factors in disease risk and hence to investigate their role as genetic factor modifiers [35]. For example, rs671 on *ALDH2* was found to be associated with TG when the interaction between SNP and alcohol consumption was considered [36]. By integrating biological knowledge with genetic pleiotropy and environmental factors, G × E interactions may provide the potential to shed light on biological processes leading to diseases and could explain ‘missing heritability’ for phenotypic variance in GWASs. And also, it may be necessary to search for additional shared genetic patterns on multiple phenotypes in larger sample sizes across diverse ethnic cohorts as well as Korean population.

## Conclusions

In summary, our results provide evidence that lipid-related loci implicated by previous GWASs also affect other cardio-metabolic traits in Korean individuals. We identified SNPs in the 12q24.12 region with ethnic-specific independent pleiotropic effects on both lipids and one or more metabolic traits. Our results may help to elucidate the East-Asian specific shared genetic background for cardio-metabolic traits that affect the risk of cardiovascular disease.

## Additional file

10.1186/s12933-016-0337-1 The direction of the allelic effect for each of three pleiotropic SNPs in the 12q24.12 region. **Figure S2.** Protein-protein interactions among three genes in the 12q24.12 region. **Table S1.** Frequency distribution of minor allele frequencies in Exome chip data. **Table S2.** Descriptive information of study subjects. **Table S3.** Replication results of known lipid-associated loci in Korean individuals. **Table S4.** Population diversity of three pleiotropic SNPs in the 12q24.12 region. **Table S5.** Evaluation of pleiotropic effect of 12q24.12 (ALDH2, rs671) genotypes. **Table S6.** Protein-protein interactions among three genes in 12q24.12 region. 
