# Supplementary material for: Evaluation of pleiotropic effects among common genetic loci identified for cardio-metabolic traits in a Korean population
Source: Cardiovasc Diabetol. 2016 Feb 1;15:20. doi: 10.1186/s12933-016-0337-1 (PMC4736473; doi:10.1186/s12933-016-0337-1)
Supplement: Supplementary file 1 — 10.1186/s12933-016-0337-1 The direction of the allelic effect for each of three pleiotropic SNPs in the 12q24.12 region. Figure S2. Protein-protein interactions among three genes in the 12q24.12 region. Table S1. Frequency distribution of minor allele frequencies in Exome chip data. Table S2. Descriptive information of study subjects. Table S3. Replication results of known lipid-associated loci in Korean individuals. Table S4. Population diversity of three pleiotropic SNPs in the 12q24.12 region. Table S5. Evaluation of pleiotropic effect of 12q24.12 (ALDH2, rs671) genotypes. Table S6. Protein-protein interactions among three genes in 12q24.12 region. [file 12933_2016_337_MOESM1_ESM.doc]

**Additional File 1**

**Evaluation of pleiotropic effects among common genetic loci identified for cardio-metabolic traits in a Korean population**

Yun Kyoung Kim, Mi Yeong Hwang, Young Jin Kim, Sanghoon Moon, Sohee Han and Bong-Jo Kim^§^

*Division of Structural and Functional Genomics, Center for Genome Sciences, National Institute of Health, Centers for Disease Control and Prevention, Chungcheongbuk-do, Korea,*

^§^Corresponding author:

Bong-Jo Kim, Ph.D.

Division of Structural and Functional Genomics, Center for Genome Science, National Institute of Health, Centers for Disease Control and Prevention, Chungcheongbuk-do, Korea, 361-951. Phone: +82-43-719-8870, Fax: +82-43-719-8908, E-mail: [kbj6181@cdc.go.kr](mailto:kbj6181@cdc.go.kr)

**Fig. S1** The direction of the allelic effect for each of three pleiotropic SNPs in the 12q24.12 region. The *x*-axis indicates the effect size calculated by meta-analysis. The *y*-axis shows eight metabolic traits. Each data point indicates the regression coefficient (beta) calculated from the meta-analysis. Error bars represent standard error. Red dashed vertical lines indicate an effect size of zero.

**Fig. S2** Protein-protein interactions among three genes in the 12q24.12 region based on data from Wiki-Pi database (http://severus.dbmi.pitt.edu/wiki-pi/). *ALDH2*, *ACAD10* and *BRAP* were functionally connected by *APP* gene. A circle in green indicates a core protein of interactions. Circles in red and purple indicate proteins of secondary and tertiary interactions, respectively.


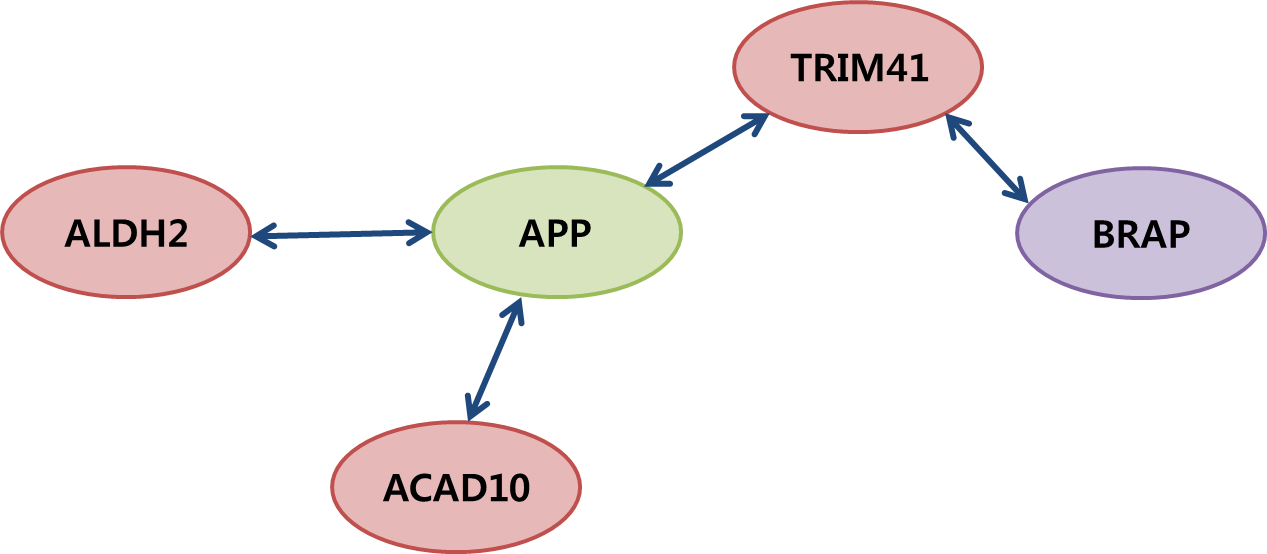


| **Table S1. Frequency distribution of minor allele frequencies in Exome chip data** | |
| --- | --- |
| MAF (%) | No. of SNP (%) |
| > 5% | 24,505 (31.6 %) |
| 1% ~ 5% | 7,078 (9.1 %) |
| 0.1% ~ 1% | 11,146 (14.4 %) |
| < 0.1% | 34,743 (44.9%) |
| Total | 77,472 |
| MAF, minor allele frequency; No., number; | |

| **Table S2. Descriptive information of study subjects** | | | |  | |  |
| --- | --- | --- | --- | --- | --- | --- |
| Traits | | KARE (n=7,524) | HEXA^*^ (n=2,786) | CAVAS  (n=3,068) | |  |
| Age | | 52.1 ± 8.81 | 53.5 ± 8.28 | 60.7 ± 8.26 | |  |
| Sex | male (%) | 3,582 (47.6) | 1,266 (45.4) | 1,352 (44.1) | |  |
|  | female (%) | 3,942 (52.4) | 1,520 (54.6) | 1,716 (55.9) | |  |
| Lipids | TG (mg/dl) | 161.4 ± 104.7 | 121.5 ± 91.4 | 153.8 ± 110.4 | |  |
|  | TC (mg/dl) | 191.5 ± 35.2 | 194.6 ± 34.1 | 200.6 ± 37.8 | |  |
|  | LDL (mg/dl) | 115.8 ± 31.5 | 117.9 ± 30.9 | 122.6 ± 32.6 | |  |
|  | HDL (mg/dl) | 44.7 ± 10.0 | 53.2 ± 12.7 | 45.6 ± 11.2 | |  |
| Blood Pressure | SBP (mmHg) | 122.2 ± 19.5 | 123.7 ± 14.7 | | - |  |
|  | DBP (mmHg) | 80.7 ± 11.8 | 78.6 ± 9.86 | - | |  |
| Glycemic traits | FPG (mg/dl) | 83.8 ± 9.64 | 90.3 ± 10.1 | 93.2 ± 10.3 | |  |
|  | HbA1c (%) | 5.55 ± 0.35 | - | - | |  |
| Obesity | BMI (kg/m^2^) | 24.6 ± 3.13 | 24.0 ± 2.89 | 24.1 ± 3.13 | |  |
|  | WHR | 0.88 ± 0.08 | 0.87 ± 0.06 | 0.90 ± 0.06 | |  |
| TG, Triglyceride; TC, Total cholesterol; LDL, Low density lipoprotein cholesterol; HDL, high density lipoprotein cholesterol; SBP, systolic blood pressure; DBP, diastolic blood pressure; FPG, fasting plasma glucose; HbA1c, Glycated hemoglobin; BMI, body mass index; WHR, waist-hip ratio; Data are shown as mean ± s.d.  ^*^ Of 3,436 HEXA subjects, we selected 2,786 subjects who had information of medication history for analyses. | | | | | | |

| **Table S3. Replication results of known lipid-associated loci in Korean individuals** | | | | | | | | | | |  |
| --- | --- | --- | --- | --- | --- | --- | --- | --- | --- | --- | --- |
| Trait | CHR | SNP | Position | Gene | Function | A1/A2 | MAF | Effect size (beta ± s.e.) | *P*_combined_ | *P*_het_(Q) |  |
| TC | 1 | 1:55509585 | 55509585 | *PCSK9* | nonsynonymous | A/G | 0.015 | -18.4 ± 1.81 | 2.33 × 10^-24^ | 0.38(1.91) |  |
|  | 2 | rs13306206 | 21242731 | *APOB* | nonsynonymous | A/G | 0.009 | 20.6 ± 2.21 | 1.24 × 10^-20^ | 0.07(5.33) |  |
|  | 2 | rs780093 | 27742603 | *GCKR* | intronic | G/A | 0.456 | -2.32 ± 0.43 | 7.94 × 10^-8^ | 0.66(0.84) |  |
|  | 5 | rs12654264 | 74648603 | *HMGCR* | intronic | T/A | 0.477 | -3.07 ± 0.43 | 8.43 × 10^-13^ | 0.52(1.31) |  |
|  | 5 | rs6882076 | 156390297 | *TIMD4* | upstream | A/G | 0.232 | -2.25 ± 0.51 | 1.13 × 10^-5^ | 0.27(2.65) |  |
|  | 9 | rs1883025 | 107664301 | *ABCA1* | intronic | A/G | 0.255 | -3.55 ± 0.49 | 5.35 × 10^-13^ | 0.71(0.69) |  |
|  | 9 | rs507666 | 136149399 | *ABO* | intronic | A/G | 0.259 | 3.01 ± 0.49 | 7.53 × 10^-10^ | 0.81(0.43) |  |
|  | 15 | rs1800588 | 58723675 | *LIPC* | intronic | A/G | 0.424 | 2.05 ± 0.44 | 2.57 × 10^-6^ | 0.98(0.04) |  |
|  | 16 | 16:57017292 | 57017292 | *CETP* | nonsynonymous | G/A | 0.046 | 6.60 ± 1.03 | 1.28 × 10^-10^ | 0.99(0.03) |  |
|  | 19 | rs7412 | 45412079 | *APOE* | nonsynonymous | A/G | 0.064 | -12.7 ± 0.88 | 9.07 × 10^-48^ | 0.56(1.15) |  |
| TG | 2 | rs1042031 | 21225753 | *APOB* | stopgain | A/G | 0.044 | 11.7 ± 3.03 | 1.10 × 10^-4^ | 0.48(1.45) |  |
|  | 2 | rs780094 | 27741237 | *GCKR* | intronic | G/A | 0.459 | -9.73 ± 1.25 | 5.42 × 10^-15^ | 0.79(0.47) |  |
|  | 7 | rs3812316 | 73020337 | *MLXIPL* | synonymous | C/G | 0.099 | -10.8 ± 2.10 | 3.12 × 10^-7^ | 0.34(2.17) |  |
|  | 8 | rs328 | 19819724 | *LPL* | stopgain | G/C | 0.123 | -17.0 ± 1.87 | 1.42 × 10^-19^ | 0.87(0.27) |  |
|  | 11 | rs174547 | 61570783 | *FADS1* | intronic | G/A | 0.319 | 7.42 ± 1.34 | 2.81 × 10^-8^ | 0.58(1.10) |  |
|  | 11 | rs1535 | 61597972 | *FADS2* | intronic | G/A | 0.319 | 7.26 ± 1.34 | 5.51 × 10^-8^ | 0.64(0.88) |  |
|  | 12 | rs3782886 | 112110489 | *BRAP* | nonsynonymous | G/A | 0.167 | -6.93 ± 1.68 | 3.53 × 10^-5^ | 0.30(2.39) |  |
|  | 12 | rs11066015 | 112168009 | *ACAD10* | intronic | A/G | 0.158 | -7.28 ± 1.71 | 2.11 × 10^-5^ | 0.34(2.15) |  |
|  | 12 | rs671 | 112241766 | *ALDH2* | nonsynonymous | A/G | 0.157 | -7.60 ± 1.72 | 9.47 × 10^-6^ | 0.36(2.06) |  |
|  | 15 | rs1800588 | 58723675 | *LIPC* | intronic | A/G | 0.424 | 5.59 ± 1.26 | 9.21 × 10^-6^ | 0.43(1.70) |  |
|  | 19 | rs7412 | 45412079 | *APOE* | nonsynonymous | A/G | 0.064 | 13.4 ± 2.55 | 1.45 × 10^-7^ | 0.20(3.24) |  |
| HDL | 2 | rs1042031 | 21225753 | *APOB* | stopgain | A/G | 0.044 | -1.29 ± 0.32 | 5.39 × 10^-5^ | 0.11(4.47) |  |
|  | 8 | rs301 | 19816934 | *LPL* | intronic | G/A | 0.205 | 1.61 ± 0.16 | 7.85 × 10^-24^ | 0.00(12.6) |  |
|  | 9 | rs1883025 | 107664301 | *ABCA1* | intronic | A/G | 0.255 | -1.33 ± 0.15 | 3.74 × 10^-19^ | 0.46(1.57) |  |
|  | 11 | 11:116707736 | 116707736 | *APOA1* | nonsynonymous | A/G | 0.038 | -1.70 ± 0.34 | 7.20 × 10^-7^ | 0.19(3.32) |  |
|  | 12 | rs3782886 | 112110489 | *BRAP* | nonsynonymous | G/A | 0.167 | -1.33 ± 0.17 | 2.97 × 10^-14^ | 0.88(0.25) |  |
|  | 12 | rs11066015 | 112168009 | *ACAD10* | intronic | A/G | 0.158 | -1.33 ± 0.18 | 7.61 × 10^-14^ | 0.83(0.38) |  |
|  | 12 | rs671 | 112241766 | *ALDH2* | nonsynonymous | A/G | 0.157 | -1.33 ± 0.18 | 1.20 × 10^-13^ | 0.73(0.63) |  |
|  | 12 | rs4759375 | 123796238 | *SBNO1* | intronic | A/G | 0.295 | 0.54 ± 0.14 | 1.69 × 10^-4^ | 0.03(6.85) |  |
|  | 12 | rs10396213 | 125296457 | *SCARB1* | nonsynonymous | G/A | 0.007 | 3.15 ± 0.76 | 3.15 × 10^-5^ | 0.01(10.5) |  |
|  | 15 | rs1800588 | 58723675 | *LIPC* | intronic | A/G | 0.424 | 1.74 ± 0.13 | 4.45 × 10^-40^ | 0.90(0.20) |  |
|  | 16 | 16:57017292 | 57017292 | *CETP* | nonsynonymous | G/A | 0.046 | 5.57 ± 0.31 | 2.29 × 10^-73^ | 0.53(1.27) |  |
|  | 17 | rs1877031 | 37814080 | *STARD3* | nonsynonymous | A/G | 0.448 | 0.48 ± 0.13 | 2.53 × 10^-4^ | 0.60(1.01) |  |
|  | 19 | rs731839 | 33899065 | *PEPD* | intronic | A/G | 0.466 | 0.49 ± 0.13 | 2.01 × 10^-4^ | 0.64(0.89) |  |
|  | 19 | rs769449 | 45410002 | *APOE* | intronic | A/G | 0.074 | -1.74 ± 0.25 | 2.54 × 10^-12^ | 0.19(3.31) |  |
| LDL | 1 | 1:55509585 | 55509585 | *PCSK9* | nonsynonymous | A/G | 0.015 | -18.6 ± 1.61 | 1.09 × 10^-30^ | 0.38(1.94) |  |
|  | 2 | rs13306206 | 21242731 | *APOB* | nonsynonymous | A/G | 0.009 | 20.1 ± 1.98 | 2.33 × 10^-24^ | 0.35(2.10) |  |
|  | 5 | rs12654264 | 74648603 | *HMGCR* | intronic | T/A | 0.477 | -2.94 ± 0.39 | 2.41 × 10^-14^ | 0.76(0.55) |  |
|  | 5 | rs6882076 | 156390297 | *TIMD4* | upstream | A/G | 0.232 | -2.18 ± 0.46 | 2.02 × 10^-6^ | 0.18(3.43) |  |
|  | 9 | rs1883025 | 107664301 | *ABCA1* | intronic | A/G | 0.255 | -1.91 ± 0.44 | 1.55 × 10^-5^ | 0.58(1.09) |  |
|  | 9 | rs507666 | 136149399 | *ABO* | intronic | A/G | 0.259 | 2.89 ± 0.44 | 4.24 × 10^-11^ | 0.88(0.26) |  |
|  | 10 | rs10761731 | 65027610 | *JMJD1C* | intronic | T/A | 0.380 | 1.43 ± 0.40 | 3.17 × 10^-4^ | 0.74(0.59) |  |
|  | 11 | rs174547 | 61570783 | *FADS1* | intronic | G/A | 0.319 | -1.82 ± 0.41 | 1.11 × 10^-5^ | 0.66(0.82) |  |
|  | 11 | rs174570 | 61597212 | *FADS2* | intronic | A/G | 0.319 | -1.82 ± 0.41 | 1.17 × 10^-5^ | 0.68(0.76) |  |
|  | 12 | rs3782886 | 112110489 | *BRAP* | nonsynonymous | G/A | 0.167 | 2.13 ± 0.52 | 3.80 × 10^-5^ | 0.93(0.14) |  |
|  | 12 | rs11066015 | 112168009 | *ACAD10* | intronic | A/G | 0.158 | 2.54 ± 0.53 | 1.50 × 10^-6^ | 0.91(0.19) |  |
|  | 12 | rs671 | 112241766 | *ALDH2* | nonsynonymous | A/G | 0.157 | 2.55 ± 0.53 | 1.53 × 10^-6^ | 0.94(0.11) |  |
|  | 12 | rs7310409 | 121424861 | *HNF1A* | intronic | A/G | 0.476 | 1.46 ± 0.39 | 1.57 × 10^-4^ | 0.15(3.73) |  |
|  | 19 | rs7412 | 45412079 | *APOE* | nonsynonymous | A/G | 0.064 | -16.9 ± 0.78 | 7.64 × 10^-104^ | 0.58(1.10) |  |
| A threshold of association significance after correction for multiple testing is *P* < 3.70 ⨯ 10^-4^. CHR, chromosome; A1/A2, minor allele/major allele; MAF, minor allele frequency; TC, Total cholesterol; TG, Triglyceride; HDL, high density lipoprotein cholesterol; LDL, Low density lipoprotein cholesterol; s.e., standard error; *P*_combined_, *P* value from meta-analyses using the results from three cohorts; *P_het_*, *P* value from test of heterogeneity; Q, Cochrane’s Q value based on chi-squared statistics. All of analyses were adjusted by age and sex using 13,378 subjects. | | | | | | | | | | |  |

| **Table S4. Population diversity of three pleiotropic SNPs in the 12q24.12 region referring from the 1000 genomes project (http://www.1000genomes.org)** | | | | | | | | | |
| --- | --- | --- | --- | --- | --- | --- | --- | --- | --- |
| Population | n | rs671 (*ALDH2*) | |  | rs11066015 (*ACAD10*) | |  | rs3782886 (*BRAP*) | |
|  |  | Allele frequency | |  | Allele frequency | |  | Allele frequency | |
|  |  | A | G |  | A | G |  | A | G |
| KOR | 14,028 | 0.157 | 0.843 |  | 0.158 | 0.842 |  | 0.833 | 0.167 |
| EAS | 1,008 | 0.174 | 0.826 |  | 0.176 | 0.824 |  | 0.825 | 0.175 |
| EUR | 1,006 | 0 | 1 |  | 0 | 1 |  | 1 | 0 |
| AFR | 1,322 | 0.002 | 0.998 |  | 0 | 1 |  | 1 | 0 |
| AMR | 694 | 0.003 | 0.997 |  | 0.003 | 0.997 |  | 0.997 | 0.003 |
| SAS | 978 | 0 | 1 |  | 0 | 1 |  | 1 | 0 |
| The comparison of allele frequencies using data from this study (KOR) and 1000 genome project (EAS, EUR, AFR, AMR and SAS). n, the number of samples; KOR, Korean individuals; EAS, East Asian Ancestry; EUR, European Ancestry; AFR, African Ancestry; AMR, American Ancestry; SAS, South Asian Ancestry | | | | | | | | | |
|  | | | | | | | | | |

| **Table S5. Evaluation of pleiotropic effect of 12q24.12 (*ALDH2*, rs671) genotypes with multiple covariates** | | | | | |
| --- | --- | --- | --- | --- | --- |
| Trait | Effect size (beta ± s.e.) | ***P* value** after adjustment for age and sex |  | after adjustment for 5 additional covariates (TG, HDL, FPG, SBP, WHR) in each model | |
|  |  |  |  | Effect size | ***P* value** |
| TG | -7.60 ± 1.72 | 9.47 × 10^-6^ |  | -9.61 ± 1.73 | 3.04 × 10^-8^ |
| LDL | 2.55 ± 0.53 | 1.53 × 10^-6^ |  | 3.43 ± 0.62 | 3.95 × 10^-8^ |
| HDL | -1.33 ± 0.18 | 1.20 × 10^-13^ |  | -1.74 ± 0.21 | 1.79 × 10^-17^ |
| FPG | -0.96 ± 0.17 | 2.00 × 10^-8^ |  | -0.62 ± 0.20 | 1.88 × 10^-3^ |
| SBP | -1.49 ± 0.32 | 2.72 × 10^-6^ |  | -0.72 ± 0.33 | 2.95 × 10^-2^ |
| DBP | -0.80 ± 0.21 | 1.09 × 10^-4^ |  | -0.43 ± 0.21 | 4.51 × 10^-2^ |
| BMI | -0.16 ± 0.05 | 2.14 × 10^-3^ |  | -0.14 ± 0.06 | 1.58 × 10^-2^ |
| WHR | -0.005 ± 0.001 | 2.87 × 10^-6^ |  | -0.005 ± 0.001 | 1.30 × 10^-4^ |
| Total cholesterol was excluded from this analysis. n = 13,378 for all traits except SBP and DBP, where n = 10,310. TG, Triglyceride; LDL, Low density lipoprotein cholesterol; HDL, high density lipoprotein cholesterol; FPG, fasting plasma glucose; SBP, systolic blood pressure; DBP, diastolic blood pressure; BMI, body mass index; WHR, waist-hip ratio; s.e., standard error; | | | | | |
|  | | | | | |

| **Table S6. Protein-protein interactions among three genes in 12q24.12 region** | | | |  |
| --- | --- | --- | --- | --- |
| Gene | Interactions | GO annotation | | |
|  |  | Molecular function | Biological process | |
| ALDH2 | 12 interactions: ALDH1A1 **APP** C14orf1 CRMP1 EIF6 FAM45B HSPD1 HSPE1 IGSF21 RPL13A SLC35F6 UNC119 | Aldehyde Dehydrogenase (NAD) Activity Aldehyde Dehydrogenase [NAD(P)+] Activity Electron Carrier Activity | Carbohydrate Metabolic Process Alcohol Metabolic Process Ethanol Catabolic Process Ethanol Oxidation Xenobiotic Metabolic Process Synaptic Transmission Neurotransmitter Biosynthetic Process Small Molecule Metabolic Process | |
| ACAD10 | 1 interaction: **APP** | Acyl-CoA Dehydrogenase Activity | Oxidation-reduction Process | |
| BRAP | 15 interactions:  BRCA1 CDC14A CENPF CNKSR1 DDB1 HMG20A HRAS NUMA1 SYNE2 **TRIM41** TRIM55 TRIM8 UBE2H USP15 USP4 | Nucleotide Binding Ubiquitin-protein Transferase Activity Protein Binding Nuclear Localization Sequence Binding Zinc Ion Binding Ligase Activity Ubiquitin Protein Ligase Activity | MAPK Cascade Ras Protein Signal Transduction Negative Regulation Of Signal Transduction Protein Ubiquitination | |
| APP | 1,995 interactions | DNA Binding Serine-type Endopeptidase Inhibitor Activity Receptor Binding Protein Binding Heparin Binding Peptidase Activator Activity Enzyme Binding Acetylcholine Receptor Binding Identical Protein Binding Transition Metal Ion Binding PTB Domain Binding Growth Factor Receptor Binding | Suckling Behavior Platelet Degranulation MRNA Polyadenylation Regulation Of Translation Protein Phosphorylation Cellular Copper Ion Homeostasis Endocytosis Response To Oxidative Stress Cell Adhesion Regulation Of Epidermal Growth Factor-activated Receptor Activity Notch Signaling Pathway Axonogenesis Blood Coagulation Mating Behavior Locomotory Behavior Axon Cargo Transport Cholesterol Metabolic Process Adult Locomotory Behavior Platelet Activation | |
| TRIM41 | 58 interactions | Protein Binding Zinc Ion Binding Ligase Activity | Protein Ubiquitination | |
